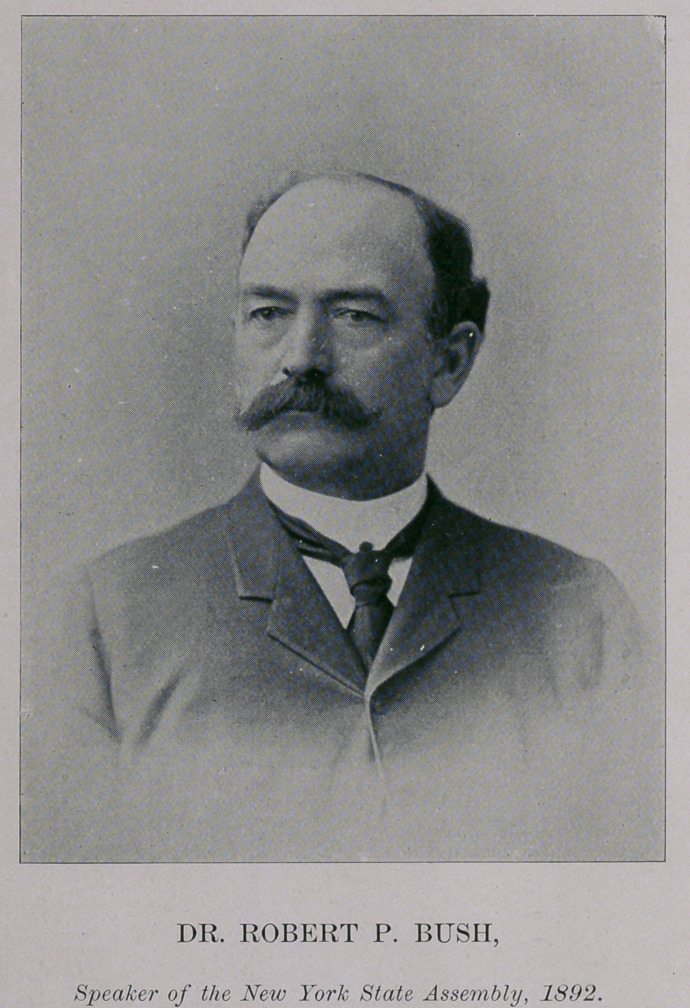# The Physician in Politics

**Published:** 1892-02

**Authors:** 


[*From the Buffalo Illustrated Express.*]

DR. ROBERT P. BUSH,

*Speaker of the New York State Assembly, 1892.*

BUFFALO MEDICAL AND SURGICAL JOURNAL

A MONTHLY REVIEW OF MEDICINE AND SURGERY.

*EDITORS:*

THOMAS LOTHROP, M. D. - - WM. WARREN POTTER, M. D.

All communications, whether of a literary or business character, should be addressed to the managing editor: 284 Franklin Street, Buffalo, N. Y.

Vol. XXXI. FEBRUARY, 1892. No. 7.

### THE PHYSICIAN IN POLITICS.

Dr. Robert P. Bush, of Horseheads, who was elected Speaker of the New York State Assembly, January 5, 1892, has the distinguished honor of being the first physician who has ever held this high office. He was chosen by the unanimous vote of his party, in caucus, to preside over the most important popular legislative body in the United States, next after the House of Representatives. Physicians throughout the State and nation should take pride in the fact that a regular practitioner of medicine, who has won an honorable name in his profession, has been accorded such distinction by his political associates.

Dr. Bush was born in Yates county in 1842, and, therefore, it is in the ripest period of his manhood that he assumes the Speaker's gavel. His preliminary education was obtained at the academies in Franklin and Cortland, and he graduated from the Medical Department of the University of Buffalo in the class of 1874. He has been in the active practice of his profession since that time, excepting during the Winters that he has spent in the Assembly, where he is now serving his seventh term.

Dr. Bush is also a veteran of the late war, with an honorable record, having served as a private in the 12th Regiment of New York Infantry during the first two years of the war. He was commissioned as Captain in the 185th Regiment of New York Infantry, September 17, 1864, promoted to Major, December 3, 1864, and mustered out with his regiment May 30, 1865. He participated in the battle of Bull Run, was at the siege of Yorktown, and in McClellan's peninsular campaign, including the seven days' fighting before Richmond, and in the battles of Antietam and Fredericksburgh. He was taken prisoner at Hatcher's Run,

October 27, 1864, but was exchanged promptly, and participated in the final campaign of the Army of the Potomac, that practically ended at Appomattox C. H., April 9, 1865.

We publish in this number an excellent picture of Speaker Bush, for which we are indebted to the Buffalo *Illustrated Express*.

## Figures and Tables

**Figure f1:**